# RNA Interference-Mediated Suppression of RAGE

**DOI:** 10.3390/ijms27209167

**Published:** 2026-10-16

**Authors:** Tatiana A. Muzaffarova, Elena M. Malinovskaya, Alexey Moskalev

**Affiliations:** Institute of Biology of Aging and Medicine of Healthy Longevity with the Clinic of Predictive Medicine, Petrovsky National Research Centre of Surgery, 119435 Moscow, Russia; tatiana.muzaffarova@mail.ru (T.A.M.); m.elena.0402@gmail.com (E.M.M.)

**Keywords:** receptor for advanced glycation end products, *AGER*, RNA interference, short hairpin RNA, gene silencing, lentiviral vectors, endothelial cells, inflammation, aging biology

## Abstract

The receptor for advanced glycation end products (RAGE/AGER) is an important mediator of inflammatory signaling and has been implicated in chronic low-grade inflammation associated with aging. Chronic low-grade inflammation (“inflammaging”) is a characteristic feature of biological aging, providing a rationale for investigating molecular regulators of inflammatory signaling such as RAGE. This study aimed to develop and validate short hairpin RNA (shRNA) constructs suppressing *AGER*/RAGE in human cell models. Five shRNA constructs targeting human *AGER* were designed, cloned into pLSL-puro, and verified by Sanger sequencing. Lentiviral vectors were produced in HEK293T cells and used to establish stable HEK293T, 786-O, and human umbilical vein endothelial cell (HUVEC) populations with *AGER* knockdown. *AGER*/RAGE expression was assessed by RT-qPCR, Western blotting, and flow cytometry. Silencing efficiency varied with the construct and the cellular model. sh1 produced the strongest suppression of *AGER*, exceeding 70% in selected models, whereas sh2 was effective in HEK293T and 786-O cells. Reduced RAGE expression was confirmed at the mRNA, protein, and cell-surface levels, identifying sh1 and sh2 as lead candidates. An *AGER*-focused workflow for lentiviral shRNA construct screening and experimental validation was established, providing a reproducible approach for generating stable cell models with reduced *AGER*/RAGE expression. Further work is required to confirm isoform specificity, assess off-target effects, and define the functional consequences of RAGE suppression.

## 1. Introduction

RAGE is a multiligand receptor capable of recognizing and binding a broad range of molecules associated with cellular damage and stress. In addition to advanced glycation end products (AGEs), its ligands include S100 proteins, HMGB1 (a prototypical alarmin), and amyloid-β peptide. It is well established that aging is accompanied by the progressive accumulation of AGEs, lipofuscin, tau protein aggregates, α-synuclein fibrils, and various damage-associated molecular patterns (DAMPs), which serve as signals of cellular stress, tissue injury, and cell death [1].

Upon binding to its ligands, RAGE initiates a complex intracellular signaling cascade that leads to the activation of transcription factors, most notably NF-κB. This, in turn, promotes the expression and release of pro-inflammatory cytokines, including tumor necrosis factor-α (TNF-α) and interleukin-6 (IL-6) [2]. Moreover, increased production of reactive oxygen species (ROS) contributes to the development of oxidative stress, and subsequent activation of immune cells further amplifies the inflammatory response. RAGE can thus be viewed as a molecular sensor that translates signals of cellular damage and stress associated with “garb-aging” into persistent, low-grade chronic inflammation [3]. Chronic, low-grade, aseptic inflammation (inflammaging) is one of the characteristic features of the aging process. The term inflammaging describes a state of persistent, typically low-level inflammation that gradually increases with age and, in turn, contributes to the biological aging of the organism. Activation of RAGE and the chronic inflammatory response associated with it contribute to the development and progression of numerous age-related diseases, including atherosclerosis, neurodegenerative disorders, and diabetic complications [4].

Studies have shown that inhibition of RAGE activity or its genetic deletion may exert protective effects. These findings open new perspectives for the development of therapeutic strategies aimed at disrupting the link between garb-aging and inflammaging. However, RAGE inhibitors designed to block ligand–receptor interactions, as well as AGE-targeted therapies, have demonstrated limited efficacy in clinical trials [5].

A promising alternative is to target *AGER* expression directly using RNA interference. In this context, a potential future strategy is selective modulation of transcripts encoding membrane RAGE while preserving soluble isoforms; however, this requires isoform-specific experimental validation. Full-length membrane-bound fl-RAGE possesses signaling activity, whereas the soluble forms sRAGE and esRAGE bind ligands and function as endogenous decoy receptors [6,7,8].

To address this challenge, the development of RNA constructs targeting sequences unique to membrane-bound RAGE transcripts, including transcript-specific splice junctions and the C-terminal coding region encoding the transmembrane and cytoplasmic domains, may be a promising approach [6,7,9].

Thus, the development of RAGE silencing strategies requires a comprehensive methodological approach that includes shRNA design and screening, assessment of isoform specificity, selection of an appropriate delivery system, and subsequent preclinical validation.

The aim of the present study was to develop shRNA constructs capable of inducing *AGER* knockdown and to evaluate their efficacy in human cell models, including human umbilical vein endothelial cells (HUVECs). HUVECs with *AGER* knockdown represent a valuable tool for fundamental research, enabling the elucidation of the role of RAGE in the regulation of vascular processes and the assessment of its potential as a therapeutic target. In our study, we examined the overall level of *AGER*/RAGE inhibition. The effect of the developed constructs on individual isoforms will be addressed in future works.

## 2. Results

### 2.1. AGER Expression Profile and Selection of Cell Models

Primer validation yielded single amplicons of the expected size, with no prominent nonspecific products detected. The efficiency of the RT-qPCR reactions, calculated from serial dilutions, ranged from 89% to 97%.

**Figure 1:**
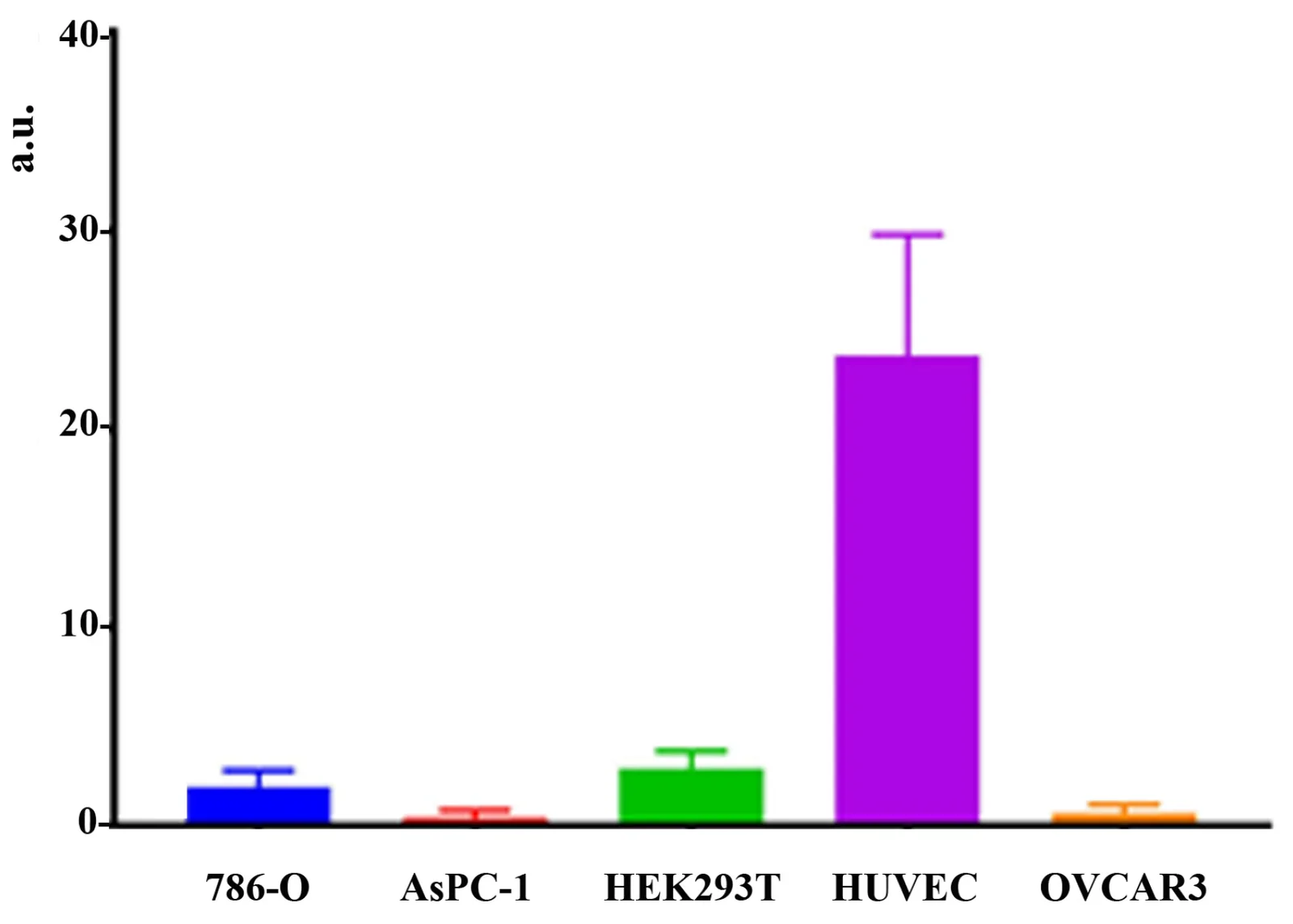
Basal *AGER* expression in human cells. Expression levels were determined by RT-qPCR and normalized to *GUSB*. Data are presented as the mean ± SD of three independent biological replicates. a.u., arbitrary units.

The highest basal *AGER* expression was observed in HUVECs, intermediate levels were detected in HEK293T and 786-O cells, and lower levels were observed in OVCAR-3 and AsPC-1 cells (Figure 1).

Based on the basal *AGER* expression profile and the complementary characteristics of the tested cell models, HUVEC, HEK293T, and 786-O cells were selected for subsequent knockdown experiments. HEK293T cells provided a highly transduction-compatible human model for methodological screening, whereas 786-O cells represented a tumor-derived renal cell model. HUVECs were selected as a vascular endothelial model because they exhibited the highest basal *AGER* expression among the tested cell types and are directly relevant to endothelial inflammatory processes. Thus, the three models were selected to assess whether shRNA efficacy was maintained across distinct cellular contexts.

The primers used in this study were designed to amplify conserved regions of the *AGER* gene shared by most isoforms. They therefore allow assessment of total *AGER* mRNA levels but are not suitable for the differential quantification of fl-RAGE and sRAGE/esRAGE transcripts. Similarly, the ab37647 antibody used for Western blotting and flow cytometry recognizes an N-terminal epitope (amino acids 1–100) that is conserved across most RAGE isoforms. Accordingly, the data presented in this study reflect the efficiency of total *AGER* knockdown and do not distinguish between individual RAGE isoforms.

### 2.2. Generation of shRNA Constructs and Establishment of Stable Transduced Cell Lines

Based on the design criteria described in Section 4.3, the hairpin sequences listed in Table 1 were constructed for *AGER* knockdown.

**Figure 2:**
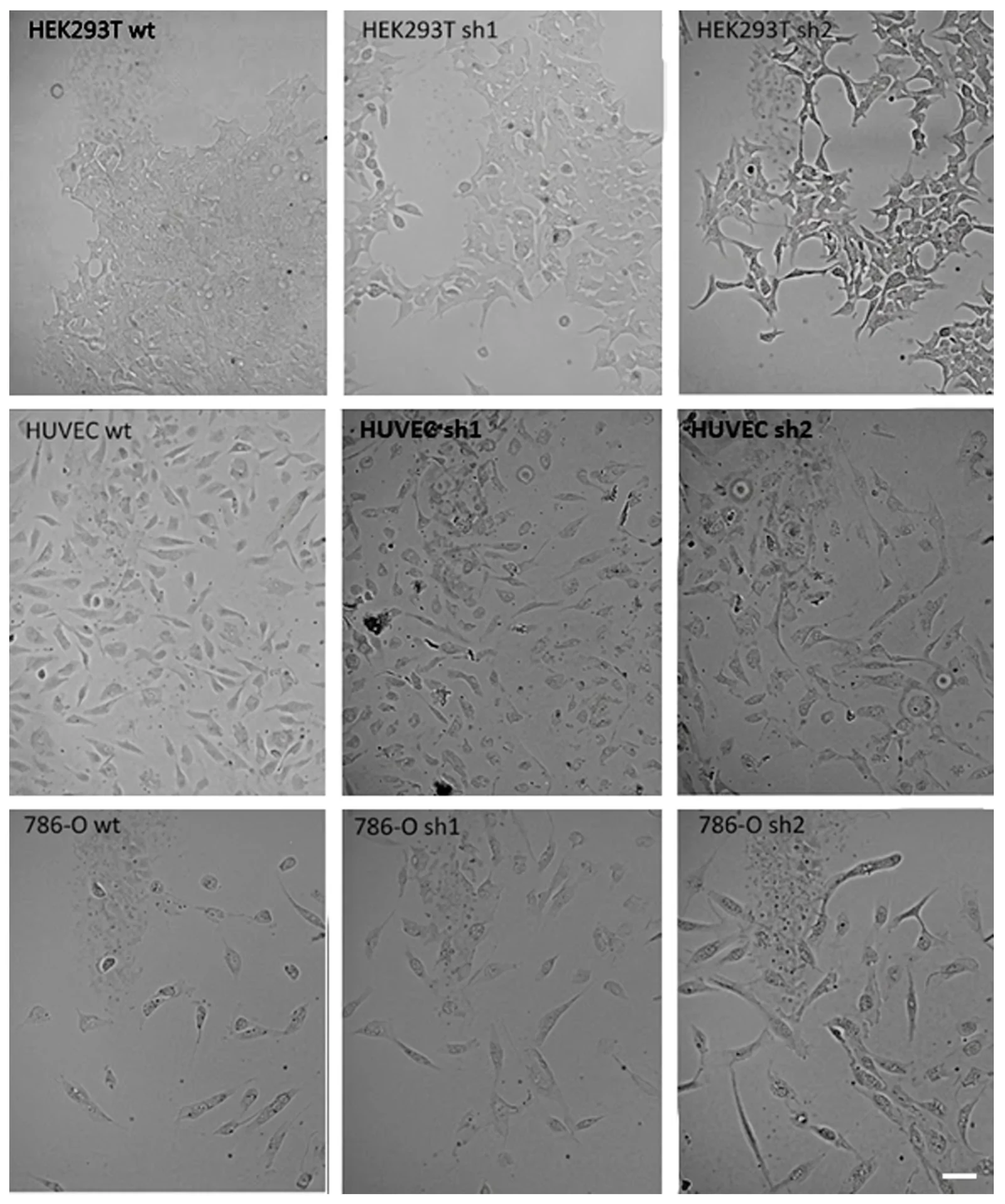
Micrographs of generated stable cell lines following puromycin selection. Representative phase-contrast images of parental (WT) and transduced HEK293T, HUVEC, and 786-O cells following puromycin selection. Scale bar, 50 µm.

HEK293T, 786-O, and HUVEC cells were transduced at a multiplicity of infection (MOI) of 2 in the presence of 8 µg/mL polybrene. After 24 h, the medium was replaced, and the transduced cells were cultured for a further 48 h before puromycin selection was initiated. Puromycin was used at 1.5 µg/mL for HEK293T, 2 µg/mL for 786-O, and 0.5 µg/mL for HUVEC cells. Selection was continued for 2–3 passages until complete elimination of the non-transduced control cells. This procedure yielded homogeneous, stable cell populations carrying the shRNA constructs (Figure 2).

### 2.3. Efficiency of AGER Knockdown at the Protein Level

Screening of three anti-RAGE antibodies (AF5309, Affinity Biosciences, Changzhou Jiangsu, China; MAB5328, Merck Millipore, Darmstadt, Germany; and ab37647, Abcam, Cambridge, UK) showed that only ab37647 produced a clear signal at the expected molecular weight of approximately 42 kDa with low background. This antibody was therefore selected for subsequent quantitative analysis.

**Figure 3:**
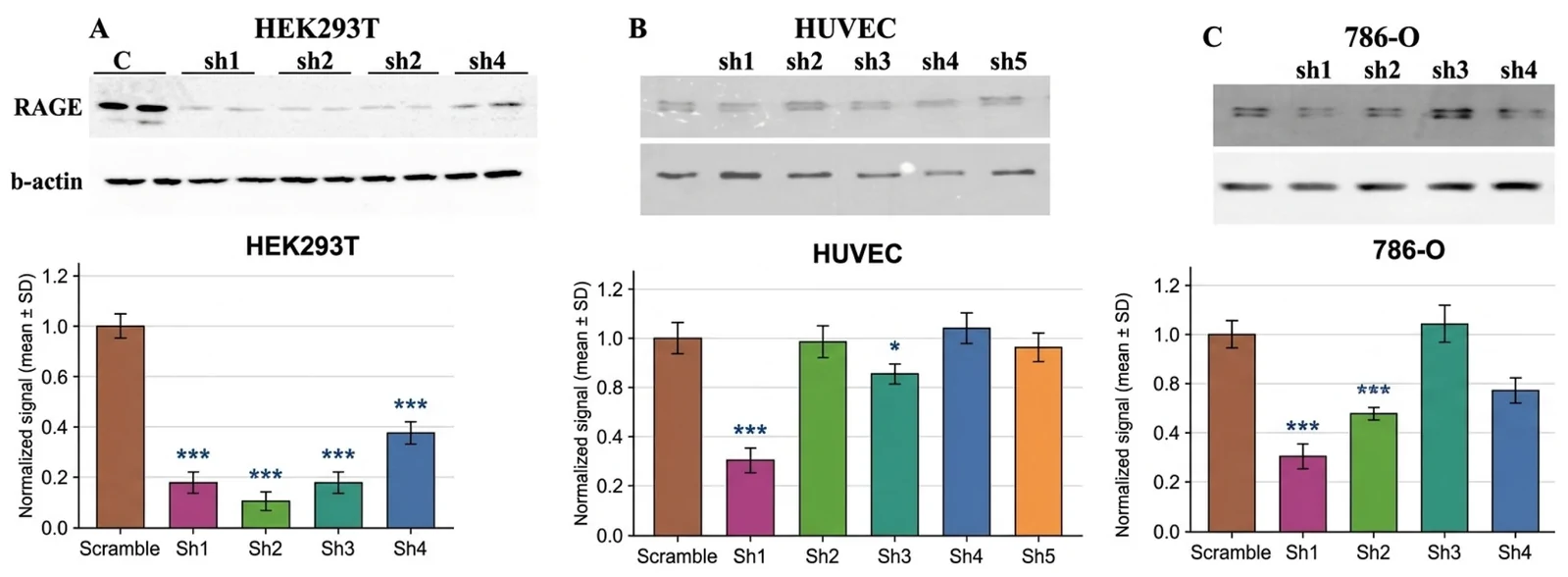
Efficiency of *AGER*/RAGE knockdown in HEK293T, HUVEC, and 786-O cell lines. (**A**) HEK293T, (**B**) HUVEC, and (**C**) 786-O cells were transduced with shRNAs targeting AGER/RAGE (sh1–sh5) alongside control groups (WT and scramble shRNA). Representative Western blot images show RAGE protein levels, with *β*-actin used as an internal loading control (**top panels**). Quantitative densitometric analysis representing normalized relative fold expression of RAGE (**bottom panels**). Data are presented as mean ± SD (*n* = 3). Statistical significance was determined using Dunnett’s multiple comparisons test against the scramble control: * *p* < 0.05, *** *p* < 0.001.

In HEK293T cells, three of the five constructs (sh1, sh2, and sh3) reduced total RAGE protein to 8–20% of control, corresponding to a knockdown of more than 70%. In 786-O cells, sh1 and sh2 lowered RAGE protein to 25–35% and 55–60% of control, respectively, whereas in HUVECs, only sh1 produced a clear reduction (25–35% of control). Construct sh4 gave a moderate reduction in HEK293T cells (33–42% of control). At the protein level, sh5 was evaluated in HUVECs only and produced no detectable change (Table 2, Figure 3).

### 2.4. Confirmation of AGER Knockdown at the mRNA Level

**Figure 4:**
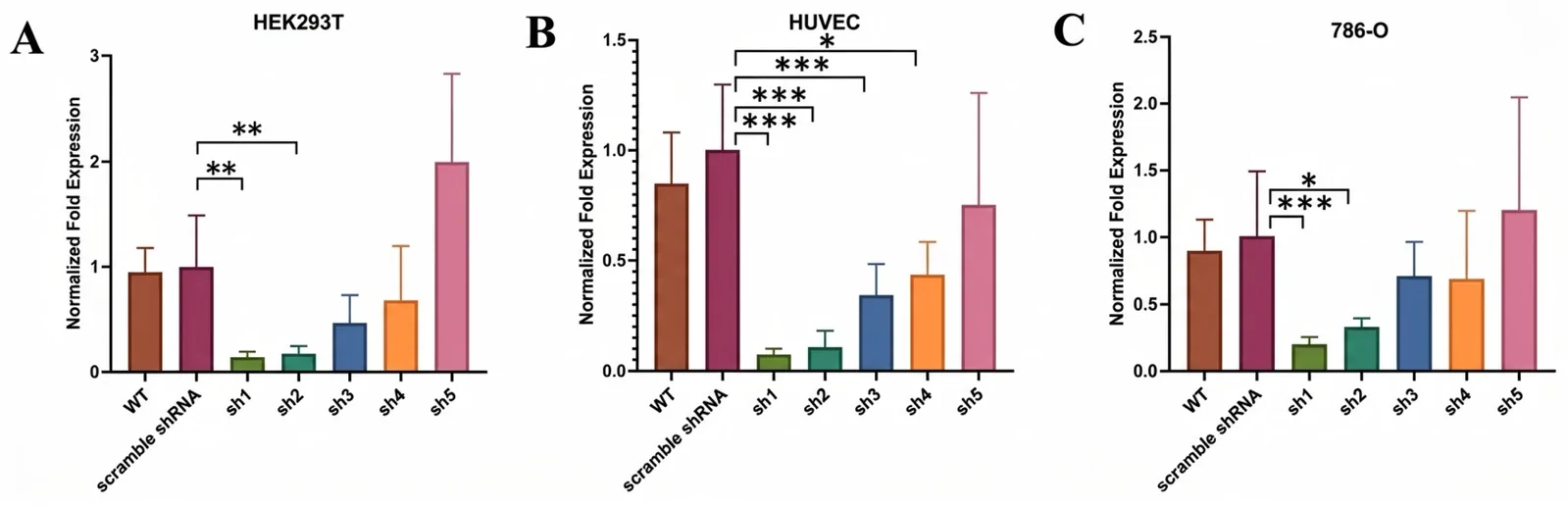
*AGER* mRNA expression in cells following transduction, assessed by RT-qPCR: (**A**) HEK293T; (**B**) HUVEC; (**C**) 786-O. Expression levels were normalized to *GUSB* and are expressed relative to the control (scrambled shRNA). Data are presented as mean ± SD of three independent biological replicates. Statistical significance was assessed by one-way ANOVA followed by Dunnett’s multiple-comparisons test. Adjusted *p*-values for comparisons with the scrambled shRNA control are indicated in the graphs. * *p* < 0.05, ** *p* < 0.01, *** *p* < 0.001.

RT-qPCR confirmed a reduction in *AGER* mRNA levels in the transduced cells; however, the magnitude of the effect varied with both the construct and the cell model (Figure 4).

For sh1 and sh2, a significant decrease in *AGER* expression (to 10–25% of control) was observed in all cell lines.

In HEK293T cells, sh1 produced the lowest *AGER* mRNA level (~10–15% of control), whereas sh2 produced the lowest RAGE protein level (~8–12% of control). Constructs sh3 and sh4 also showed substantial mRNA knockdown.

The sh1 construct demonstrated the most consistent knockdown efficiency across all three cell lines.

### 2.5. Assessment of RAGE Surface Expression by Flow Cytometry

**Figure 5:**
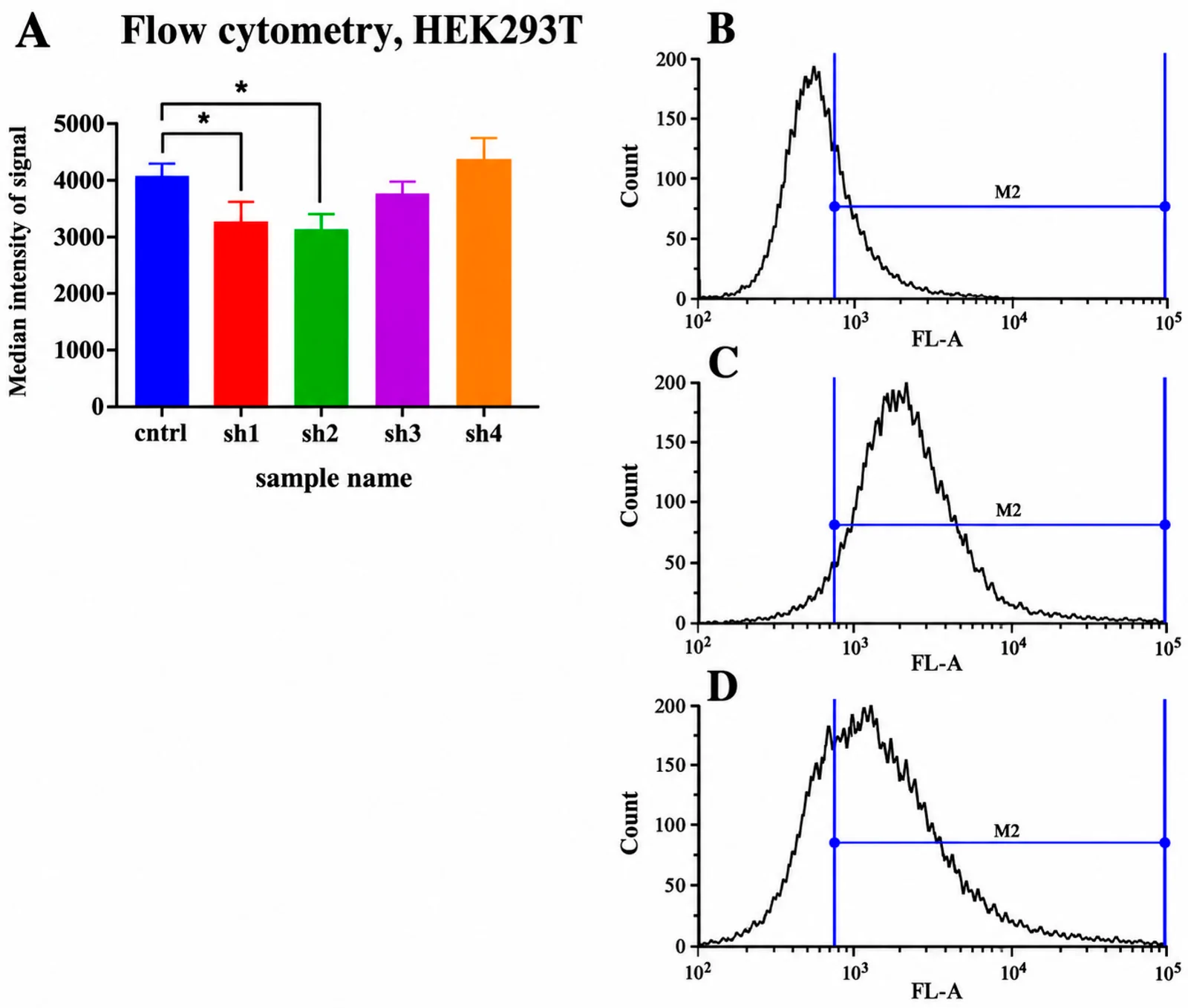
Relative surface expression of RAGE in HEK293T cells assessed by flow cytometry: (**A**) median fluorescence intensity of the analyzed samples (*, differences versus control are significant at *p* < 0.05); (**B**) fluorescence intensity histogram in the PE channel for control cells stained with the secondary antibody only; (**C**) control cells stained with both primary and secondary antibodies; (**D**) knockdown cells transduced with the sh1 construct. Data are presented as the mean ± SD of three independent biological replicates.

Flow cytometry further demonstrated a significant reduction in surface RAGE expression in HEK293T cells transduced with the sh1 and sh2 constructs (Figure 5).

## 3. Discussion

In this study, shRNA constructs targeting various regions of *AGER* mRNA were developed. This approach enabled the assessment of the feasibility and reproducibility of shRNA-mediated *AGER* expression silencing across different cell types. The overall level of *AGER*/RAGE inhibition was determined. However, selectively targeting the full-length membrane-bound RAGE isoform is preferable, as it allows for the preservation of soluble RAGE isoforms. Among the generated constructs, the Sh1 construct warrants particular attention; it targets a sequence within exon 10 of the *AGER* gene, thereby ensuring the selective silencing of the full-length membrane-bound RAGE isoform. Evaluating the selective effect on the full-length RAGE transcript represents an important next step in the research. The results demonstrate that RNA interference is a technically feasible approach for reducing *AGER* expression and enables the generation of stable cell models with substantial knockdown. Importantly, a leading construct, sh1, was identified, with reductions in *AGER* levels exceeding 70% relative to control in several experimental models. The concordant reduction in the *AGER* signal at the mRNA, protein, and cell-surface levels further supports the selection of this construct as the most promising candidate.

At the same time, the differences observed between cell lines indicate that shRNA efficacy cannot be extrapolated directly from one cellular model to another. For example, in HEK293T cells, the sh2 construct reduced the protein level to about 10% of control, whereas in HUVECs, it did not lower RAGE protein at all. This finding has important practical implications for the development of therapeutic strategies, highlighting the need for preliminary evaluation of shRNA efficacy in disease-relevant cell types rather than relying solely on readily transduced cell lines such as HEK293T.

An important limitation of the present study is that the specificity of the most active constructs, particularly sh1 and sh2, was not directly evaluated at the transcriptome level. Sequence-based screening and the use of independent *AGER*-targeting constructs reduce but do not eliminate the possibility of off-target effects. Therefore, the present data support efficient *AGER* knockdown but should not be interpreted as definitive evidence that all downstream effects of sh1 or sh2 are exclusively *AGER*-dependent. Further validation should include systematic analysis of predicted off-target transcripts and, where functional phenotypes are investigated, rescue experiments using an shRNA-resistant *AGER* expression construct. Such experiments would provide stronger evidence for the causal specificity of the observed phenotypes.

In our study, HUVECs were among the cell lines subjected to *AGER* knockdown. HUVECs with reduced *AGER* expression represent a valuable tool for fundamental research, enabling the elucidation of the role of RAGE in the regulation of vascular processes and the assessment of its potential as a therapeutic target for vascular dysfunction associated with age-related diseases. Such an approach is consistent with the principles of precision medicine, in which therapeutic strategies are tailored to the specific tissue type and pathological context.

The cell type- and construct-specific differences observed in the present study may result from several biological and technical factors. At the cellular level, differences in *AGER* transcript abundance, alternative splicing patterns, transcript stability, and the relative contribution of individual RAGE isoforms may influence the apparent efficiency of a given shRNA. In addition, cell type-specific differences in lentiviral transduction, integration, H1 promoter activity, intracellular RNA-processing capacity, and the activity of the RNA-induced silencing complex may contribute to variation in knockdown efficiency. These factors may be particularly relevant when comparing HEK293T cells with HUVECs and 786-O cells, which differ substantially in their biological characteristics and basal *AGER* expression.

At the construct level, differences in target-site accessibility within the *AGER* transcript may affect the efficiency with which the shRNA-derived guide RNA can engage its target. Local RNA secondary structure, sequence composition, thermodynamic properties, and the abundance or stability of the targeted transcript may all influence RNAi efficiency. The observed differences between sh1 and sh2 provide an example of this effect: sh2 produced strong protein suppression in HEK293T cells but had little effect on RAGE protein in HUVECs, despite reducing *AGER* mRNA in both models. This observation suggests that transcript-level silencing alone does not necessarily predict the magnitude of protein-level suppression.

The discrepancy between mRNA and protein knockdown may additionally reflect differences in RAGE protein turnover and post-translational stability. Thus, a reduction in *AGER* mRNA may precede or exceed the corresponding reduction in cellular RAGE protein, particularly when the pre-existing protein has a relatively long half-life. Conversely, differences in protein synthesis, degradation, and membrane trafficking between cell types may modify the relationship between transcript abundance and steady-state protein levels. These possibilities remain mechanistic hypotheses and will require direct experimental testing.

Of particular interest is the relationship between knockdown efficiency at the mRNA and protein levels. For sh1, good concordance was observed, with *AGER* levels reduced to approximately 10–25% of control at both levels. For sh2, a correspondence between the reduction in mRNA and protein levels was also observed in HEK293T cells. For the other constructs, the reduction in *AGER* mRNA levels did not translate into a decrease in protein levels. This mRNA–protein discordance may reflect multilayered post-transcriptional regulation of the RAGE pathway; possible explanations include the rate of protein turnover, post-translational modifications that increase protein stability, translation efficiency, and RNA stability. Functional validation of shRNA constructs should therefore also include parameters characterizing the status of signaling pathways, such as NF-κB activation, pro-inflammatory cytokine production, oxidative stress, cell migration, and tissue-specific phenotypes.

A particularly important direction for future work is to address isoform specificity. The concept of selectively suppressing the membrane-bound form of RAGE while preserving the soluble sRAGE and esRAGE isoforms appears biologically more rational than complete suppression of *AGER*, because soluble RAGE isoforms can act as ligand-binding decoy receptors [7]. RNA interference has the potential to target fl-RAGE selectively. However, the RT-qPCR and Western blotting approaches used in the present study primarily assess total *AGER* expression and do not allow definitive conclusions regarding the preservation of individual soluble isoforms. The next stage of the study should therefore include isoform-specific primers, analysis of alternative splicing variants, and, where necessary, separate quantification of membrane-bound and soluble RAGE.

Recent studies have identified the RAGE/DIAPH1 axis as an additional signaling module that may represent a target for suppressing RAGE signaling. According to a recent review, the cytoplasmic domain of RAGE interacts with the protein DIAPH1 [8], and disruption of this interaction may also attenuate pathological signaling mediated by fl-RAGE [9]. A combination of RNA interference and blockade of the RAGE/DIAPH1 interaction might therefore produce a more pronounced inhibitory effect on fl-RAGE.

The technology used to deliver RNA constructs is of great importance for RAGE knockdown in vivo [10]. The biological activity of an RNAi construct cannot be considered independently of its tissue distribution, intracellular delivery, stability, and toxicity [11]. Direct RAGE silencing has also been explored using non-viral RNA delivery systems: a recent study demonstrated that PEG-modified cationic liposomes co-delivering apigenin and RAGE siRNA reduced RAGE expression and inflammatory signaling and provided protection against myocardial ischemic injury in a rat model [12]. This result shows that suppression of RAGE can be achieved not only via viral delivery systems but also through alternative approaches. The lentiviral platform used in the present study should therefore be regarded primarily as an effective experimental system for screening and validating candidate sequences rather than as the only potential delivery strategy. The potential of AAV, lentiviral, and non-viral delivery platforms should be evaluated in the context of the target tissue, the required duration of the therapeutic effect, immunogenicity, and the ability to control shRNA expression levels. For chronic conditions, the controllability and safety of long-term gene silencing are particularly important. Progression to in vivo studies should therefore be preceded by systematic evaluation of dose dependence, tissue tropism, target specificity, and potential off-target effects.

From a methodological standpoint, this study presents a methodological workflow targeting the *AGER* gene, comprising the design of target sequences, cloning, lentiviral vector production, the generation of stable cell models, and a multi-level assessment of knockdown efficiency. A systematic comparison of five independent shRNA constructs across three human cell models provides an experimental basis for identifying constructs capable of reproducibly silencing *AGER* and for evaluating how knockdown efficiency depends on the cellular context. The proposed approach may facilitate further functional studies on *AGER*/RAGE biology, including in the context of inflammation and aging processes.

Evaluating knockdown efficiency at multiple levels not only confirms the reduction in target gene expression but also helps identify potential compensatory mechanisms or off-target effects associated with interference with cellular signaling pathways. Such multilayered validation increases the reliability of data interpretation and strengthens the conclusions regarding the role of RAGE in the cellular processes under investigation. It also provides a foundation for further evaluation of potential therapeutic strategies aimed at modulating RAGE activity. The validated shRNA constructs may provide useful experimental tools for subsequent investigation of RAGE-dependent inflammatory signaling and age-associated cellular phenotypes. However, the present study does not establish whether *AGER* suppression modifies inflammaging or other age-related phenotypes, and such conclusions require dedicated functional experiments.

**Limitations of the study.** The present study was primarily methodological in nature and focused on preclinical in vitro development.

Direct experimental assessment of potential off-target effects of the shRNA constructs was not performed in the present study. Therefore, the specificity of sh1 and sh2 requires additional validation before these constructs can be considered suitable for definitive mechanistic or therapeutic studies.

The functional consequences of *AGER* knockdown for inflammatory signaling pathways were not investigated directly, and the issue of isoform specificity remains unresolved. In addition, the differences in construct efficacy observed across cell models require further independent and reproducible validation. These limitations should be taken into account when interpreting the findings and designing subsequent in vivo studies.

## 4. Materials and Methods

### 4.1. Primer Design and Assessment of Basal AGER Expression

Primers for RT-qPCR were designed using NCBI Primer-BLAST (*AGER* forward, 5′-GTCCAACTACCGAGTCCG-3′; *AGER* reverse, 5′-CGACTGCAGTGTGAAGAG-3′). *GUSB* (beta-glucuronidase) was used as the reference gene (*GUSB* forward, 5′-CGTGGTTGGAGAGCTCATTTGGAA-3′; *GUSB* reverse, 5′-ATTCCCCAGCACTCTCGTCGGT-3′). Amplification was performed on a CFX96 real-time PCR detection system (Bio-Rad, Hercules, CA, USA) with 5X qPCRmix-HS SYBR (Evrogen, Moscow, Russia), using 45 cycles of denaturation at 95 °C for 10 s, annealing at 58 °C for 15 s, and elongation at 72 °C for 15 s. Relative expression was calculated using the ΔΔCt method.

RNA was isolated using ExtractRNA reagent (Evrogen, Moscow, Russia). Reverse transcription was performed in a 20 µL reaction with 300–600 ng of DNase-treated RNA using Mint reverse transcriptase (Evrogen, Moscow, Russia) and random hexamer primers (Sileks, Moscow, Russia). Quantitative PCR was run in four technical replicates, and biological samples were obtained in three independent replicates.

Basal *AGER* expression was examined in the following human cells: OVCAR-3 (ovarian carcinoma), 786-O (renal cell carcinoma), HEK293T (embryonic kidney), AsPC-1 (pancreatic adenocarcinoma), and HUVEC (human umbilical vein endothelial cells).

### 4.2. Cell Culture

All cell lines were obtained from ATCC (Manassas, VA, USA): OVCAR-3 (HTB-161), 786-O (CRL-1932), HEK293T (CRL-11268), AsPC-1 (CRL-1682), and HUVEC (CRL-1730), and were cultured as described by the supplier. The HUVEC line was at passage 5 at the time of transduction and at passage 9 at the completion of selection. Phase-contrast images were acquired on a Zeiss AxioVert 200 microscope (Carl Zeiss, Oberkochen, Germany).

### 4.3. Design and Cloning of shRNA Constructs

Five candidate target sequences were selected from the *AGER* mRNA sequence (NM_001136.5) using predefined sequence-selection criteria rather than by random selection. Candidate sites were selected to minimize the presence of known polymorphisms, to provide high predicted target specificity based on BLAST analysis, and to maintain a GC content of approximately 50%. Multiple independent target sites were included because the efficiency of RNA interference can be strongly dependent on the local sequence context and accessibility of the target region and therefore cannot be reliably predicted from a single candidate sequence. The five candidates were subsequently evaluated experimentally in different human cell models.

This computational assessment was used for candidate selection but was not considered sufficient to exclude off-target effects experimentally.

The canonical CACGTG loop was introduced to form the hairpin. Oligonucleotides were annealed and cloned into the pLSL-puro vector after digestion with BamHI (ER0051, Thermo Scientific, Waltham, MA, USA) and EcoRI (ER0271, Thermo Scientific, Waltham, MA, USA); 5′ and 3′ overhangs compatible with the restriction sites of the vector were added for this purpose. The pLSL-puro vector was kindly provided by Dr. A.V. Lipatova (Engelhardt Institute of Molecular Biology, Russian Academy of Sciences, Moscow, Russia) and was adapted for expression under the control of the H1 promoter. The correctness of the inserts was confirmed by restriction analysis and Sanger sequencing (Applied Biosystems 3500 Series Genetic Analyzer, Thermo Fisher Scientific, Waltham, MA, USA). Alignment of the sequences obtained was performed using SnapGene Viewer 8.2.3(GSL Biotech LLC, San Diego, CA, USA).

### 4.4. Production of Lentiviral Preparations and Generation of Stable Cell Lines

Lentiviral vectors were produced in HEK293T cells using a four-plasmid system consisting of pLSL-puro-shRNA and the packaging plasmids pGAG-Pol, p-REV, and p-VSVG (ViraPower Lentiviral Expression System, Invitrogen, Carlsbad, CA, USA). Transfection was performed by lipofection with Lipofectamine 3000 (Thermo Fisher Scientific, Waltham, MA, USA). Viral supernatants were collected at 36, 48, 60, and 72 h, filtered through a 0.45 µm membrane, and stored at −80 °C. Functional viral titers were determined by serial dilution followed by puromycin selection.

HEK293T, 786-O, and HUVEC cells were transduced at an MOI of 2 in the presence of 8 µg/mL polybrene (H9268, Sigma-Aldrich, St. Louis, MO, USA). After medium replacement, the cells were cultured for 48 h and then subjected to puromycin selection (puromycin dihydrochloride, A1113803, Thermo Fisher Scientific, Waltham, MA, USA). Stable cell populations were generated for subsequent analysis of *AGER* expression.

### 4.5. Assessment of AGER Expression

*AGER* mRNA levels were assessed by RT-qPCR with normalization to *GUSB*. Protein expression was analyzed by Western blotting. Cell lysates were prepared in RIPA lysis and extraction buffer (89900, Thermo Scientific, Waltham, MA, USA) supplemented with a protease inhibitor cocktail (Halt Protease Inhibitor Cocktail, 78410, Thermo Scientific, Waltham, MA, USA). Equal amounts of protein (40 µg) were separated by SDS-PAGE and transferred onto PVDF membranes (0.45 µm, 88585, Thermo Scientific, Waltham, MA, USA). After transfer, membranes were blocked for 1 h at room temperature in 5% skim milk in TBST. RAGE was detected with an anti-RAGE antibody (1:1000, ab37647, Abcam, Cambridge, UK), a rabbit polyclonal antibody validated for human, mouse, rat, and bovine samples and for Western blotting and flow cytometry. An antibody against β-actin (sc-81178, Santa Cruz Biotechnology, Dallas, TX, USA) was used for signal normalization. The secondary antibody was a goat anti-rabbit blotting-grade horseradish peroxidase conjugate (1:1000; 1706515, Bio-Rad, Hercules, CA, USA). Precision Plus Protein Unstained Standards (Bio-Rad, Hercules, CA, USA) were used to estimate protein molecular weights. The antibody produced a clear specific signal with minimal nonspecific background. Band intensities were analyzed using the Gel Doc XR+ system with Image Lab 5.2.1 image capture and analysis software (Bio-Rad, Hercules, CA, USA) and ImageJ J2(NIH, Bethesda, MD, USA). Uncropped images of all blots are provided in Figures S1–S3.

Cell-surface RAGE expression was assessed by flow cytometry without cell permeabilization. Briefly, live cells were stained with the primary anti-RAGE antibody (ab37647, 1:1000) for 1 h at room temperature, followed by an Alexa Fluor 555-conjugated donkey anti-rabbit IgG secondary antibody (A-31572, Thermo Fisher Scientific, Waltham, MA, USA; 1:1000) in 0.5% BSA in PBS. The secondary antibody is a highly cross-adsorbed polyclonal donkey anti-rabbit IgG (H + L) antibody conjugated to Alexa Fluor 555.

A total of 30,000 events per sample were analyzed on the basis of PE-channel fluorescence using a BD LSRFortessa cell analyzer (BD Biosciences, Franklin Lakes, NJ, USA). Data were analyzed using BD FACSDiva v9.0 software (BD Biosciences, Franklin Lakes, NJ, USA) and Flowing Software 2.2 (Turku Centre for Biotechnology, University of Turku and Åbo Akademi University, Turku, Finland).

### 4.6. Preparation of Plasmid DNA for Further Studies

The validated plasmid construct was amplified in an *E. coli* culture and purified using sequential alkaline lysis, extraction, precipitation, and purification steps. A total of 1.2 mg of plasmid DNA was obtained. DNA concentration and purity were assessed spectrophotometrically from the A260/A280 ratio. The resulting plasmid DNA preparation was intended for subsequent expansion of the preclinical studies.

### 4.7. Statistical Analysis

All quantitative experiments were performed using three independent biological replicates, with four technical replicates per biological replicate. Statistical analyses were performed using the biological replicates as the experimental units; technical replicates were averaged before statistical testing.

Data are presented as the mean ± standard deviation (SD) and were analyzed with GraphPad Prism 8.0.2.263 (GraphPad Software, Boston, MA, USA). Relative RAGE mRNA expression was calculated using the (2^−ΔΔCt^) method, with *GUSB* serving as the reference gene and the scramble group used as the calibrator. Statistical analyses were performed on ΔCt values. Differences among experimental groups were assessed separately for each cell line using one-way analysis of variance (ANOVA), followed by Dunnett’s multiple comparisons test to compare each shRNA-treated group with the scramble control. Data are presented as mean ± standard deviation (SD). A *p*-value < 0.05 was considered statistically significant.

## 5. Conclusions

This study established and experimentally validated an *AGER*-focused workflow for lentiviral shRNA-mediated suppression of *AGER*/RAGE.

shRNA constructs were designed and cloned, lentiviral preparations were generated, and stable human cell models were established. Comprehensive evaluation by RT-qPCR, Western blotting, and flow cytometry identified sh1 as the most promising candidate for *AGER* knockdown, with reductions in RAGE expression exceeding 70% in some experimental models. These findings demonstrate the technical feasibility of sustained *AGER* modulation using shRNA.

A next step is to test whether isoform-selective suppression of membrane RAGE transcripts can be achieved while preserving soluble isoforms. This will require isoform-specific assays, off-target analysis, and functional validation of altered RAGE signaling. Such studies would provide the necessary foundation for subsequent preclinical in vivo evaluation.

Further studies should determine whether suppression of AGER/RAGE alters inflammatory signaling and cellular phenotypes relevant to inflammaging. Such studies should include direct assessment of pathways such as NF-κB activation, pro-inflammatory cytokine production, oxidative stress, and cellular senescence, as well as appropriate disease- and age-relevant experimental models.

## Figures and Tables

**Table 1:** shRNA sequences targeting human *AGER*.

No.	Name	shRNA Sequence
1	shRNA-AGER1	gatccg ATCAGGGCTGGGAACTCTAG cacgtg CTAGAGTTCCCAGCCCTGAT tttttg
2	shRNA-AGER2	gatccg AATGGAAACTGAACACAGGC cacgtg GCCTGTGTTCAGTTTCCATT tttttg
3	shRNA-AGER3	gatccg GAAGCCAGAAATTGTAGATTC cacgtg GAATCTACAATTTCTGGCTTC tttttg
4	shRNA-AGER4	gatccg AAGAAACCACCCCAGCGGCT cacgtg AGCCGCTGGGGTGGTTTCTT tttttg
5	shRNA-AGER5	gatccg AATAAGGTGGGGACATGTGT cacgtg ACACATGTCCCCACCTTATT tttttg
6	Scramble shRNA	gatccg AGTTCATCTGCACCACCGGCA cacgtg TGCCGGTGGTGCAGATGAACT tttttg

All constructs were cloned into the pLSL-puro vector and verified by sequencing. The resulting plasmids were then used for the production of lentiviral preparations. Functional viral titers ranged from 4 × 10^6^ to 3 × 10^7^ TU/mL.

**Table 2:** Efficiency of *AGER*/RAGE knockdown in the transduced cell lines.

Cell Line	Group	Western Blot Signal/Densitometry (Mean ± SD)	*p* vs. Scramble	Significance	Relative RAGE Expression (Mean ± SD)	*p* vs. Scramble	Significance
HEK293T	Scramble	1.000 ± 0.046	—	—	1.040 ± 0.355	—	—
	Sh1	0.175 ± 0.043	<0.0001	***	0.171 ± 0.119	<0.001	***
	Sh2	0.100 ± 0.037	<0.0001	***	0.224 ± 0.103	<0.001	***
	Sh3	0.175 ± 0.043	<0.0001	***	0.456 ± 0.218	0.1403	ns
	Sh4	0.375 ± 0.045	<0.0001	***	0.714 ± 0.282	0.74	ns
	Sh5	—	—	—	2.058 ± 0.564	0.2777	ns
786-O	Scramble	1.000 ± 0.055	—	—	1.044 ± 0.388	—	—
	Sh1	0.300 ± 0.050	<0.0001	***	0.127 ± 0.050	<0.0001	***
	Sh2	0.575 ± 0.025	<0.0001	***	0.289 ± 0.120	<0.01	**
	Sh3	1.040 ± 0.075	0.7840	ns	0.734 ± 0.419	0.6372	ns
	Sh4	0.750 ± 0.050	0.0035	**	0.602 ± 0.042	0.4677	ns
	Sh5	—	—	—	1.247 ± 0.631	0.9921	ns
HUVEC	Scramble	1.000 ± 0.062	—	—	1.026 ± 0.275	—	—
	Sh1	0.300 ± 0.050	<0.0001	***	0.099 ± 0.038	<0.0001	***
	Sh2	0.985 ± 0.065	0.9520	ns	0.167 ± 0.031	<0.0001	***
	Sh3	0.850 ± 0.040	0.0142	*	0.346 ± 0.096	<0.0001	***
	Sh4	1.040 ± 0.060	0.8840	ns	0.536 ± 0.022	0.03249	*
	Sh5	0.960 ± 0.060	0.7810	ns	0.745 ± 0.103	0.45	ns

NOTE. Data are presented as mean ± SD. Statistical significance is indicated relative to the corresponding scramble control: * *p* < 0.05; ** *p* < 0.01; *** *p* < 0.001; ns, not significant (*p* ≥ 0.05).

## Data Availability

The original contributions presented in this study are included in the article and in the Supplementary Materials. The raw RT-qPCR data, Western blot densitometry, and flow-cytometry data supporting the reported results are available from the corresponding author upon reasonable request.

## Supplementary Materials

The following supporting information can be downloaded at: https://www.mdpi.com/article/10.3390/ijms27209167/s1.

## Abbreviations

The following abbreviations are used in this manuscript:

AGEs	advanced glycation end products
AGER	advanced glycation end-product specific receptor (gene)
ANOVA	analysis of variance
DAMPs	damage-associated molecular patterns
esRAGE	endogenous secretory RAGE
fl-RAGE	full-length (membrane-bound) RAGE
GUSB	beta-glucuronidase
HUVEC	human umbilical vein endothelial cells
IL-6	interleukin-6
MOI	multiplicity of infection
RAGE	receptor for advanced glycation end products
RNAi	RNA interference
ROS	reactive oxygen species
RT-qPCR	reverse transcription quantitative polymerase chain reaction
SD	standard deviation
shRNA	short hairpin RNA
siRNA	small interfering RNA
sRAGE	soluble RAGE
TNF-α	tumor necrosis factor-α
TU	transducing units
WT	wild type
ns	not significant
