# Supplementary material for: RNA Interference-Mediated Suppression of RAGE
Source: Int J Mol Sci. 2026 Oct 16;27(20):9167. doi: 10.3390/ijms27209167 (PMC13705484; doi:10.3390/ijms27209167)
Supplement: Supplementary file 1 [file ijms-27-09167-s001.zip › ijms-4605157-supplementary-highlight.pdf]

Supplementary Materials

RNA Interference-Mediated Suppression of RAGE

Tatiana A. Muzaffarova, Elena M. Malinovskaya and Alexey Moskalev

This file contains the uncropped images of all Western blot membranes used to prepare Figure 3 of the main text. In each panel the boxed area indicates the region reproduced in the main figure. Membranes were imaged with the Gel Doc XR+ system (Bio-Rad, Hercules, CA, USA); where visible, the numbers at the left margin indicate the positions of the molecular weight markers (kDa).

(A)

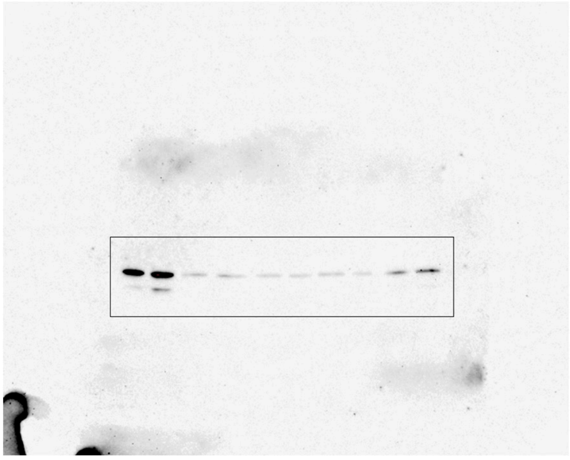

(B)

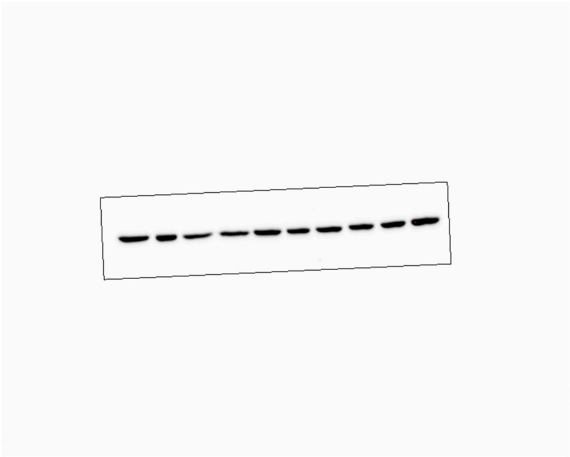

Figure S1. Uncropped Western blot images for HEK293T cells. (**A**) Membrane probed with the anti-RAGE antibody (ab37647); (**B**) membrane probed with the anti-β-actin antibody (sc-81178). The boxed area in each panel corresponds to the cropped image shown in Figure 3A of the main text.

(A)

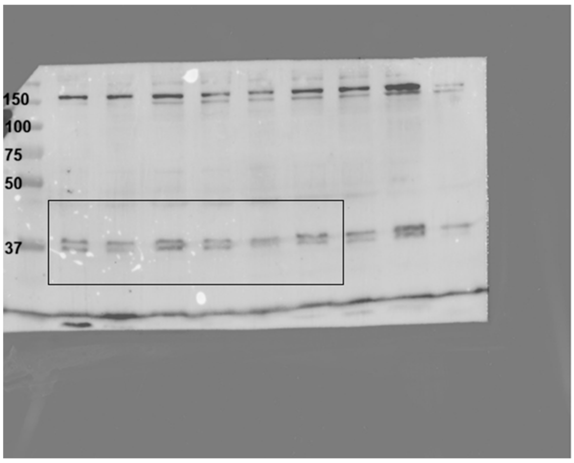

(B)

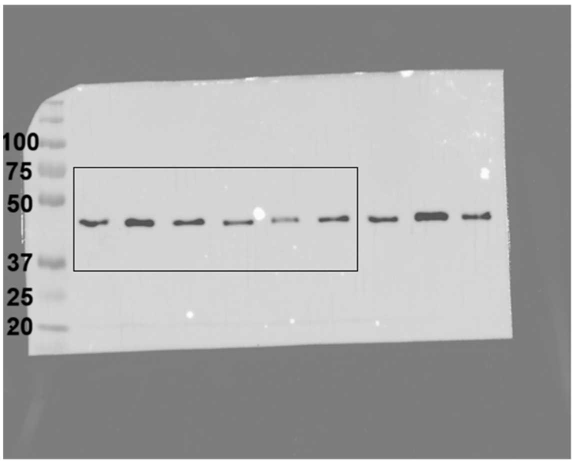

Figure S2. Uncropped Western blot images for HUVEC cells. (**A**) Membrane probed with the anti-RAGE antibody (ab37647); (**B**) membrane probed with the anti-β-actin antibody (sc-81178). The boxed area in each panel corresponds to the cropped image shown in Figure 3B of the main text.

(A)

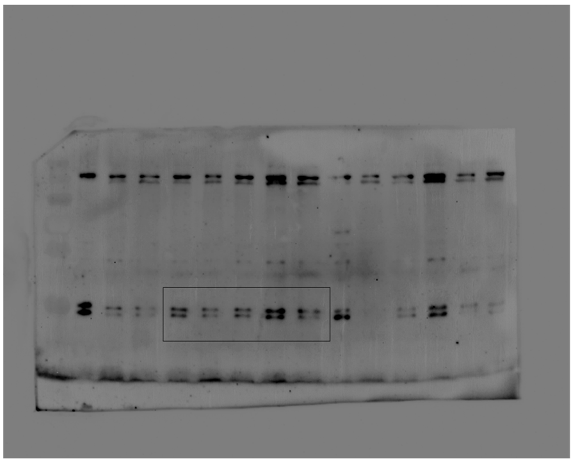

(B)

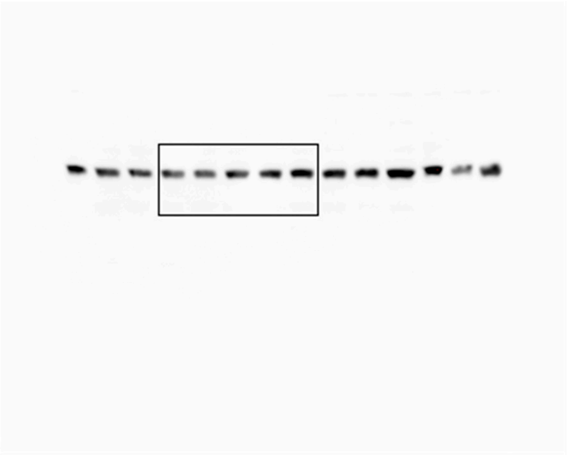

Figure S3. Uncropped Western blot images for 786-O cells. (**A**) Membrane probed with the anti-RAGE antibody (ab37647); (**B**) membrane probed with the anti-β-actin antibody (sc-81178). The boxed area in each panel corresponds to the cropped image shown in Figure 3C of the main text.

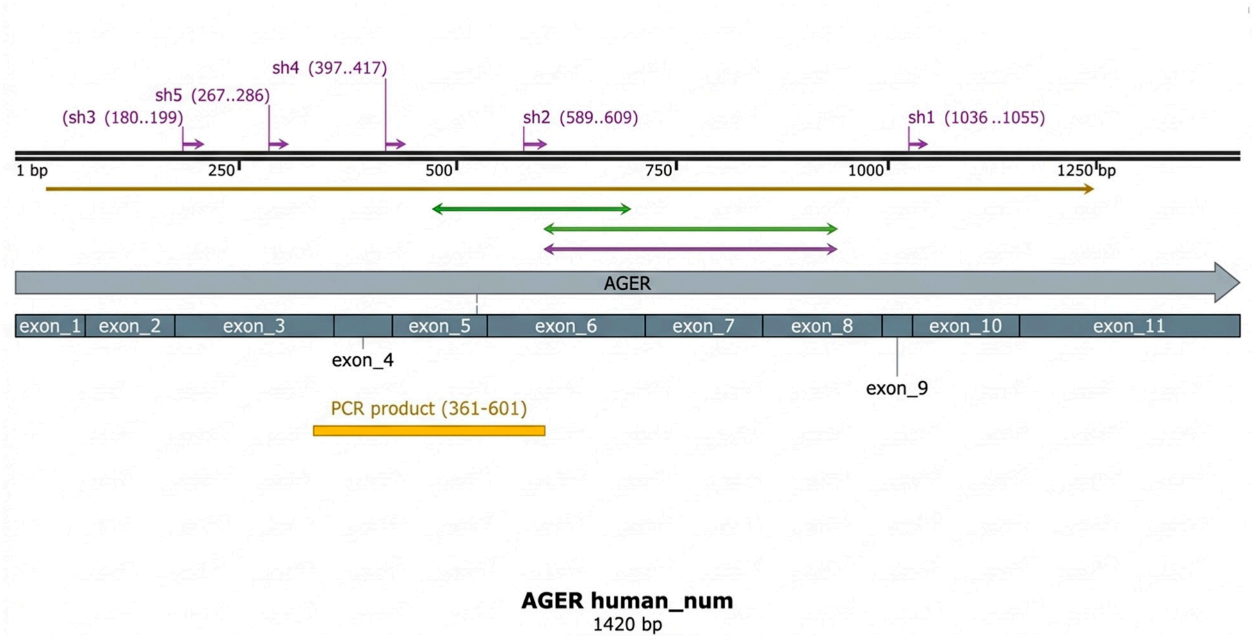

Figure S4. Schematic representation of the human *AGER* transcript and positions of the shRNA target sequences and RT-qPCR amplicon. The diagram shows the major transcript regions of *AGER* (NM_001136.5), the locations of the five shRNA target sequences (sh1–sh5), and the region amplified by the AGER RT-qPCR primers. The diagram is not drawn to scale.The reference transcript used for shRNA design in the manuscript is NM_001136.5, which is the reviewed canonical *AGER* transcript in the current NCBI annotation.
